# Production of a New Plant-Based Milk from *Adenanthera pavonina* Seed and Evaluation of Its Nutritional and Health Benefits

**DOI:** 10.3389/fnut.2018.00009

**Published:** 2018-02-12

**Authors:** Israel Sunmola Afolabi, Irene Chiamaka Nwachukwu, Chinemelum Sandra Ezeoke, Ruth Chineme Woke, Olawunmi Adebisi Adegbite, Tolulope Dorcas Olawole, Olubukola C. Martins

**Affiliations:** ^1^Biochemistry Department, College of Science and Technology, Covenant University, Ota, Nigeria; ^2^Lagos State University Teaching Hospital (LASUTH) Complex, Lagos State Drug Quality Control Laboratory, Ikeja, Nigeria

**Keywords:** food quality, milk, processing, vitamins, minerals, adenosine triphosphate synthase, acetylcholinesterase, health

## Abstract

A new plant milk was discovered from the seed of *Adenanthera pavonina*. The physicochemical and nutritional properties of the new pro-milk extract were assessed, and their biochemical effects were compared with those of soy bean extracts. Eleven groups of three albino rats each were used to assess the health benefits of the pro-milk. Groups were separately administered 3.1, 6.1, and 9.2 µl/g animal wt. pro-milk extract from *A. pavonina* seed, 6.1 µl/g animal wt. milk extract from soybean, and 6.1 µl/g animal wt. normal saline for 7 or 14 days. The “baseline” group consisted of those sacrificed on day 0. Among the physical properties considered, the pro-milk from *A. pavonina* had significantly higher (*P* < 0.05) hue color value and significantly lower (*P* < 0.05) L* than that from soy bean did. The pro-milk from *A. pavonina* had a significantly higher (*P* < 0.05) level of protein (36.14 ± 0.12%), Ca (440.99 ± 0.93 mg/l), Mg (96.69 ± 0.03 mg/l), K (190.41 ± 0.11 mg/l), Na (64.24 ± 0.24 mg/l), and Cu (0.55 ± 0.24 mg/l), and a significantly lower (*P* < 0.05) level of Mn (0.04 ± 0.01 mg/l) and vitamins A (undetectable), C (1.87 ± 0.01 mg/100 g), and E (0.12 ± 0.01 mg/100 g) compared to those of soy milk. The daily consumption of the pro-milk extract from *A. pavonina* for 14 days significantly reduced (*P* < 0.05) Ca^2+^-adenosine triphosphate synthase (Ca^2+^-ATPase) at low dose (3.1 µl/g animal wt.), but significantly increased (*P* < 0.05) Mg^2+^-ATPase at high dose (9.2 µl/g animal wt.). Daily administration of the *A. pavonina* extract for 14 days caused a significant reduction (*P* < 0.05) in acetylcholinesterase activity in the liver, intestine, heart, and kidney, suggesting that the pro-milk may facilitate ions transportation across the membrane. The pro-milk offers promising beneficial effects for patients with neurological diseases, as well as supporting general health owing to the high protein and mineral content. Vitamins fortification is recommended during production.

## Introduction

Strategies, such as transforming the current underutilized and/or poisonous plant resource to their edible forms and biotransforming the present waste to nutrients or sources of nutrients, are required to meet the nutritional needs of the increasing global population (1). Plants had largely been useful for ages as a source of nourishing substances. Important nourishing substances derived from plants include palm wine, which is useful for curing eye diseases and chicken pox (2, 3); palm oil, which provides strong antioxidant such as vitamin A (4); Carica papaya seed oil and leaf of *Solenostemon monostachyus*, which serve as sources of nutrients and bioactive compounds with health benefits like antisickling (5); and milk from soybean, coconut, rice, and almond, which provide nutrients to sustain human and livestock health (6–9). Different types of plant milks, including tigernut milk, peanut milk, lupin milk, cowpea milk, oat milk, rice milk, corn milk, walnut milk, sesame milk, sunflower milk, and amaranth milk, have recently been identified and classified under cereal-based, legume-based, nut-based, seed-based, and pseudo-cereal-based categories depending on their sources (10–12). Nutritional drinks such as these plant milks are usually consumed by people to satisfy taste or thirst, and certain people consume it daily as a tea supplement to obtain a healthy diet (13, 14). Presently, there is no recommended dosage for these commercial drinks due to the unpredictable pattern of drinking commercial milks by consumers. The consumption of nutritional drinks depends on the satisfaction of the consumer regarding the nutritional content (13).

Milk is originally the first natural source of nutrients for mammals especially for humans at childbirth (15). Breast milk was programmed by nature to basically supply lactose and calcium, protein, vitamins B12, riboflavin, and minerals such as potassium, phosphorus, magnesium, and zinc that are required to sustain a newborn child till the time of weaning (16, 17). The macronutrient, calcium, helps to build strong bones, prevents the development of bone diseases like osteoporosis, and plays a role in cell signaling, energy metabolism, blood pressure regulation, muscle contraction, and hormonal action. A newborn child is also suited to feed on breastmilk for 2–3 years prior to when the genes involved in lactose production are downregulated (18, 19). Lactase persistence or lactose tolerance occurs due to mutation in the human genome leading to adults being able to consume milk thereby increasing the demand for milk, and increasing the price of milk required to sustain the young generation to adulthood (18). Efforts to provide a solution for these problems have led to the production and consumption of milk from other animals including cow, buffalo, and goats (16, 20, 21). Recent findings have implicated that the consumption of milk from these animals led to the development of health complications such as body pains, deformed body structure in both adult male and female, early breast development among under-aged children due to hormonal imbalance generated by interference of other hormones meant for these lower animals, in humans (22). Certain cultural and religious practices in some countries discourage the use of these animals as a source of milk, thereby reducing the consumption of their milk further (10). As a result, the attention of the research community has shifted to plant milk from soybeans, rice, and almond that are generally low in total and saturated fat and do not contain cholesterol (2, 6, 23).

*Adenanthera pavonina* L. (family: Fabaceae; subfamily: Mimosoideae) is a leguminous food tree with seed that is roasted and consumed by people living in some region along the Pacific Ocean (24, 25). It is also a good source of phenolic antioxidant with antiviral, antidiabetic, antibacterial, antifungal, antiparasitic, anticonvulsant, and anti-inflammatory activity among numerous other health benefits (26, 27). The seeds of *A. pavonina* are highly nutritious and rich in essential amino acids and non-protein amino acids (28, 29), including gamma methylene glutamic acid and gamma methylene glutamine. The kernel contains a pale yellow fat, which consists of palmitic, arachidic, lignoceric, oleic, linoleic, eicosanoic, and octacosanoic acids. Stigasterol and its glucoside, dulcitol, and a polysaccharide are also present in the kernel (30–32). The presence of isovitexin, β-glucopyranoside, kaempferol, and quercetin also contribute to the bioactivity of *A. pavonina* (33). There was no report on *A. pavonina* serving as source of milk. This study was therefore aimed to develop a method for the production of milk from the seed of *A. pavonina* and to assess the suitability of its consumption by monitoring its physicochemical and *in vivo* biochemical properties in relation to membrane integrity and neurological status to compare it to the already established and commercialized milk extracted from soy beans.

## Materials and Methods

### Seed Collection

*A. pavonina* seeds were harvested at Covenant University, Canaanland Ota, Ogun State, and as identified (the identifications number) and labeled within the surrounding, while the soy bean seeds [Glycine max (L.) Merr.] were purchased at a local Otta market. The seeds were identified by Dr. M. F. Popoola of the Applied Biology and Biotechnology Department of Covenant University. The voucher specimen number (FHI107848) for *A. pavonina* was deposited earlier in Forest Research Institute of Nigeria (Ibadan).

### Preparation of the Pro-Milk Aqueous Extract from *A. pavonina* and Soy Bean Seeds

#### *A. pavonina* Aqueous Extract Preparation

*A. pavonina* seeds (700 g) were cooked using a microwave oven (Currys Essentials C17MW10) for 2–3 min, and soaked in water (5 l) for 48 h. The water was decanted and the seeds were rewashed with water for about 4–5 times until the water becomes clear of the characteristic red color. The seed was peeled by a braising method in which the seeds were placed in between two hands, and this method was based on the local processing method for cowpea. The peeled seed coat was separated and removed by decanting the excess water in which they were floating. The resultant peeled seeds were blended with water (1:4 w/v) using an Emel blender (EM-242) for 2–3 min. The filtrate, which should be chocolate milk-like color, was collected by filtering the blended mixture through muslin. The collected filtrate was thereafter allowed to stand for 15–20 min to obtain the milky colored suspension (pro-milk extract) used for this study. A portion of the peeled *A. pavonina* seeds were oven dried at 30°C for 20 min, and blended into a flour (A-Pav. flour) for color comparison with that of similarly treated flour (soy flour) obtained from the peeled seeds during the extraction of soy milk.

#### Extraction of Aqueous Extract (Soy Milk) from Soy Beans

The same procedure above was used for the extraction of the soy milk (Soy-M), except that the soaking period was 10 h, and it was not subjected to the decantation after the last 15–20 min standing before collecting the milk.

### Animal Treatment

A total of 33 male albino rats of about 150–180 g each were purchased from animal house of University of Agriculture, Abeokuta, Ogun State, Nigeria. The rats were divided into 11 groups of 3 rats, and allowed to acclimatize for 4 weeks during which food and water were made available *ad libitum*. They were later subjected to 24-h fasting at the end of the first and second week before being sacrificed under diethylether anesthetization. This study was carried out in accordance with the recommendations of the Guidelines for Ethical Conduct in the Care and Use of Nonhuman Animals in Research, Committee on Animal Research and Ethics (CARE). The protocol was approved (reference number CU/BIOSCRECU/BIO/2015/010) by the Biological Sciences Research Ethics Committee, Covenant University, Ota, Ogun State, Nigeria, prior to beginning the study. Twelve hours alternate light and dark cycles, at 24–28°C, 58 ± 5% RH. were maintained in the animal house where the experimental rats were kept.

### Experimental Design and Administration of Extracts

The extracts were orally administered with the aid of cannula directly into the alimentary canal of the rats. The rats in the “7Pav-M(3.1)” group were administered daily with 0.5 ml pro-milk extract from *A. pavonina* seed for 7 days, while those in the “14Pav-M(3.1)” group were similarly administered 0.5 ml for 14 days. The rats in the “7Pav-M(6.1)” group were administered daily with 1.0 ml pro-milk extract from *A. pavonina* seed for 7 days, while those in the “14Pav-M(6.1)” group were similarly administered 1.0 ml for 14 days. The rats in the “7Pav-M(9.2)” group were administered daily with 1.5 ml pro-milk extract from *A. pavonina* seed, while those in the “14Pav-M(9.2)” were similarly administered daily with 1.5 ml for 14 days. In addition, the rats in the “7Soy-M” group were daily administered 1.0 ml pro-milk extract from soy bean seed for 7 days, while those in “14Soy-M” group were similarly administered daily with 1.0 ml for 14 days. The two control groups were daily administered 1.0 ml of normal saline (0.9% w/v) for 7 days (C-7), and for 14 days (C-14). The normal saline served as a control to ensure the changes noted during experimentation were due the effect of administration of the pro-milk extracts alone, and not due to the method of administration of extracts using canola. The “baseline” group were the rats sacrificed on day 0 in order to assess the status of the rats just before the commencement of feeding. The choice of numbers (7 or 14) added before the abbreviation for each group were to indicate the days of administration, and the numbers within bracket to indicate the level of dosage (3.1, 6.1, and 9.2 µl/g animal wt. equivalent for 0.5, 1.0, and 1.5 ml, respectively) administered. The dosage was carefully selected after considering the maximum amount (1.5 ml) that the rat could conveniently consume without stress as nutritional drinks often consumed in large volumes depending on the satisfaction of the consumer with the nutritional content. The volumes, 0.5 and 1.0 ml, were thereafter considered at equal interval to represent low and moderate consumption group. All treatments and further analysis were performed independently on each rat.

#### Organs Preparation for Analysis

At the end of the each treatment phase (7 or 14 days), the organs (brain, heart, kidney, liver, and small intestine) were excised: the organs were rinsed in normal saline, 0.2 g of the organs were weighed and homogenized using 1.8 ml of 0.9% normal saline with the exception of the brain which was homogenized using 1.8 ml 0.1 M phosphate buffer. The homogenate was then centrifuged at 4,000 × *g* for 10 min and stored at −20°C prior to using it in the acetylcholinesterase and cytochrome P450 assay.

### Analyses

#### Physicochemical Properties

The color of the seed and the extracts at various processing stages were determined using Hunter Lab MiniScan EZ (Model MS521062/Serial no. 40009), the Hue value (*h*°) and color index (CI) for the milk extracts were determined using the formula described by Nagle, Intani (34), and the color difference (ΔE*) between the two milk extracts was also calculated using the following formula described by Addala et al. (35).

(1) $$h{}^{\circ}=\text{Arc tan }{(}^{*}b{/}^{*}a)$$

where, *h*° is the hue angle; a* and b* are the color values in CIE L*a*b* color space

(2) $$\text{CI}=(1,000\times a^{*})/(L\times b^{*})$$

(3) $$\Delta E^{*}={\left[{\left(L_{\text{s}}^{*}{}_{}-L_{\text{a}}^{*}\right)}^{2}+{\left(a_{\text{s}}^{*}-a_{\text{a}}^{*}\right)}^{2}+{\left(b_{\text{s}}^{*}-b_{\text{a}}^{*}\right)}^{2}\right]}^{1/2}$$

where subscript “s” indicates soy milk extract; and subscript “a” indicates *A. pavonina* seed milk extract. For the *L* value which is “*L*” (Lightness) axis, 0 is black and 100 is white. Similarly for “*a*” (red-green) axis, positive values are red, negative values are green, and 0 is neutral. For “*b*” (blue-yellow) axis, positive values are yellow, negative values are blue, and 0 is neutral.

The density of the extracts was determined by measuring the equivalent weight for 5.0 ml of the extracts and dividing such weight by their equivalent volume.

#### Total Protein Estimation

Total protein content of the extracts was assayed using micro-Kjeldhal method (36), while that of the organs extracts was determined spectrophotometrically using Folin-Lowry method (37). The absorbance in the Folin-Lowry method was measured at 600 nm and the corresponding concentration was quantified using a standard graph.

#### Minerals

The extracts were digested using the AOAC wet-ash method described by Boyer (38), and the mineral content was determined using the Atomic Absorption Spectrophotometer (Shimadzu, model AA-7000) following the method described by de Ruig (39). A total of 5.0 g of sample was weighed into a beaker and 20 ml of aqua regia (mixture of conc. HCl: HNO_3_; 3:1) was added. The mixture was boiled gently until the volume of the aqua regia was reduced to 5.0 ml. Subsequently, 20 ml of distilled water was added, and the heating continued until the organic matter was completely digested. The digested sample was filtered, and the volume was made up to 100 ml with distilled water using a volumetric flask. The mineral content of this resultant solution was measured using the use of Atomic Absorption Spectrometer with a hollow cathode lamp using prepared standards as appropriate for the different minerals to be analyzed. Samples were aspirated and the mean signal responses were recorded at each of the element respective wavelength.

#### Preparation of Extracts for High Performance Liquid Chromatography (HPLC) Analysis

An aliquot of 1.0 g of the liquid extracts were vigorously shaken in 50.0 ml mobile phase (absolute methanol) using a flask shaker for 10 min, and filtered prior to been injected into the HPLC.

#### Vitamins Analysis Using HPLC

An aliquot of 10 µl of the filtrate for each of the milk extract was injected into the HPLC system (Agilent 1200) using an appropriate column Zorbax Eclipse XDB-C18 (4.6 × 150 mm, 5 µm) for analysis of fat-soluble vitamins, and Hypersil BDS C18 (4.6 × 125 mm, 5 µm) for the analysis of vitamins C. Both analyses were conducted at a flow rate (2.0 ml/min), wavelength of 280 nm, and mobile phase of methanol (absolute) for the fat-soluble vitamins and a wavelength of 220 nm and a mobile phase of acetonitrile:water pH 2.16 (1:99) for vitamin C. Vitamin A (Metagenics, 250,000 U/g), vitamin C (BDH, 99.89% purity), vitamin D3 (Sigma Aldrich, 1 mg/ml ampoule), and vitamin E (Sigma Aldrich, 96% purity) were used as standard during analysis. The concentration of the pro-milk extracts was calculated from the result obtained for each vitamin standards determined using the following formula.

$$P=\frac{\text{Area of sample}\times \text{Concentration of standard }(\text{mg/ml})\times \text{Purity of the standard }(\%)}{\text{Area of standard }(\text{mg/ml})\times \text{Concentration of sample }(\text{mg/ml})}.$$

#### Assay for Acetylcholinesterase and Cytochrome P450

Acetylcholinesterase and cytochrome P450 were measured by enzyme-linked immunosorbent assay using ELISA kit (Hangzhou Eastbiopharm). The assay was performed following the instructions provided by the manufacturer. The optical density was read at a wavelength of 450 nm using an enzyme reader. The same procedure was used for the determination of cytochrome P450 (except that acetylcholinesterase antibody was used to replace the same amount cytochrome P450 antibody). The corresponding activity of acetylcholinesterase and cytochrome P450 were quantified using a standard graph following the manufacturer’s instruction.

#### Assay for Adenosine Triphosphate Synthase (ATPase)

The activity of ATPase was determined using the procedure described by Elekwa, Monanu (40). The assay of Na^+^-K^+^, Mg^2+^ and Ca^2+^ ATPases were carried out on the erythrocyte membrane of rat whole blood cells. The absorbances were read at 725 nm using a spectrophotometer, while the corresponding enzyme activity in molar (M) of inorganic phosphate (Pi)/ml blood sample was quantified using a standard graph.

### Statistical Analysis

One factor randomized complete block design was used for this experiment. Data were analyzed using analysis of variance MegaStat statistical software package supplied by Dataville Solutions Ltd. (Lagos, Nigeria). Results with a *P* value of <0.05 were considered statistically significant, and they were subjected to a Student’s *t*-test using the same statistical software. The results for each analysis were obtained in replicated and were expressed as mean ± SD.

## Results

### Physicochemical Properties of the Extracts

The hue color value of the pro-milk extract from *A. pavonina* was significantly higher (*P* < 0.05) but the L* was significantly lower (*P* < 0.05) compared to those of the extract from soy bean. This difference in L* resulted in overall color difference of 15.33 ± 1.67 (Table 1). The processing of both the *A. pavonina* seed and the soy bean seed into flour also resulted in significant increase (*P* < 0.05) of the L*, b*, and hue values, but led to the significant reduction (*P* < 0.05) of their “a*” and the CI values (Table 1).

**Table 1:** Physicochemical qualities of milk extract from *Adenanthera pavonina* and soy bean.

	A Pav-M	Soy-M	A Pav-seed	Soy-seed	A Pav-flour	Soy flour
Density (g/ml)	1.010 ± 0.014^a^	0.995 ± 0.007^a^				
Color						
*L*	57.53 ± 1.48^a^	72.78 ± 1.82^b^	44.76 ± 0.43^c^	62.51 ± 0.26^d^	50.37 ± 0.71^e^	68.09 ± 1.23^f^
a*	0.04 ± 0.04^a^	−0.53 ± 0.02^a^	13.28 ± 2.74^b^	6.15 ± 0.11^c^	6.81 ± 0.61^c^	5.32 ± 0.23^c^
b*	9.73 ± 0.52^a^	8.54 ± 0.53^a^	4.39 ± 0.74^b^	17.61 ± 0.00^c^	12.97 ± 1.96^d^	26.56 ± 1.56^e^
Hue value	1.57 ± 0.00^a^	−1.51 ± 0.00^b^	0.32 ± 0.01^c^	1.24 ± 0.00^d^	1.09 ± 0.02^e^	1.37 ± 0.01^f^
Color index (CI)	0.08 ± 0.07^a^	−0.85 ± 0.05^a^	67.28 ± 2.60^b^	5.59 ± 0.11^c^	10.50 ± 0.74^d^	2.97 ± 0.04^e^
Color difference between the two extracts (ΔE*)	15.33 ± 1.67					

*ND, not detected*.

*Values = mean ± SD (n = 5); data within each row with different superscript alphabet are statistically different (P < 0.05)*.

### Nutritional Qualities of Pro-Milk Extract from *A. pavonina*

**Figure 1:**
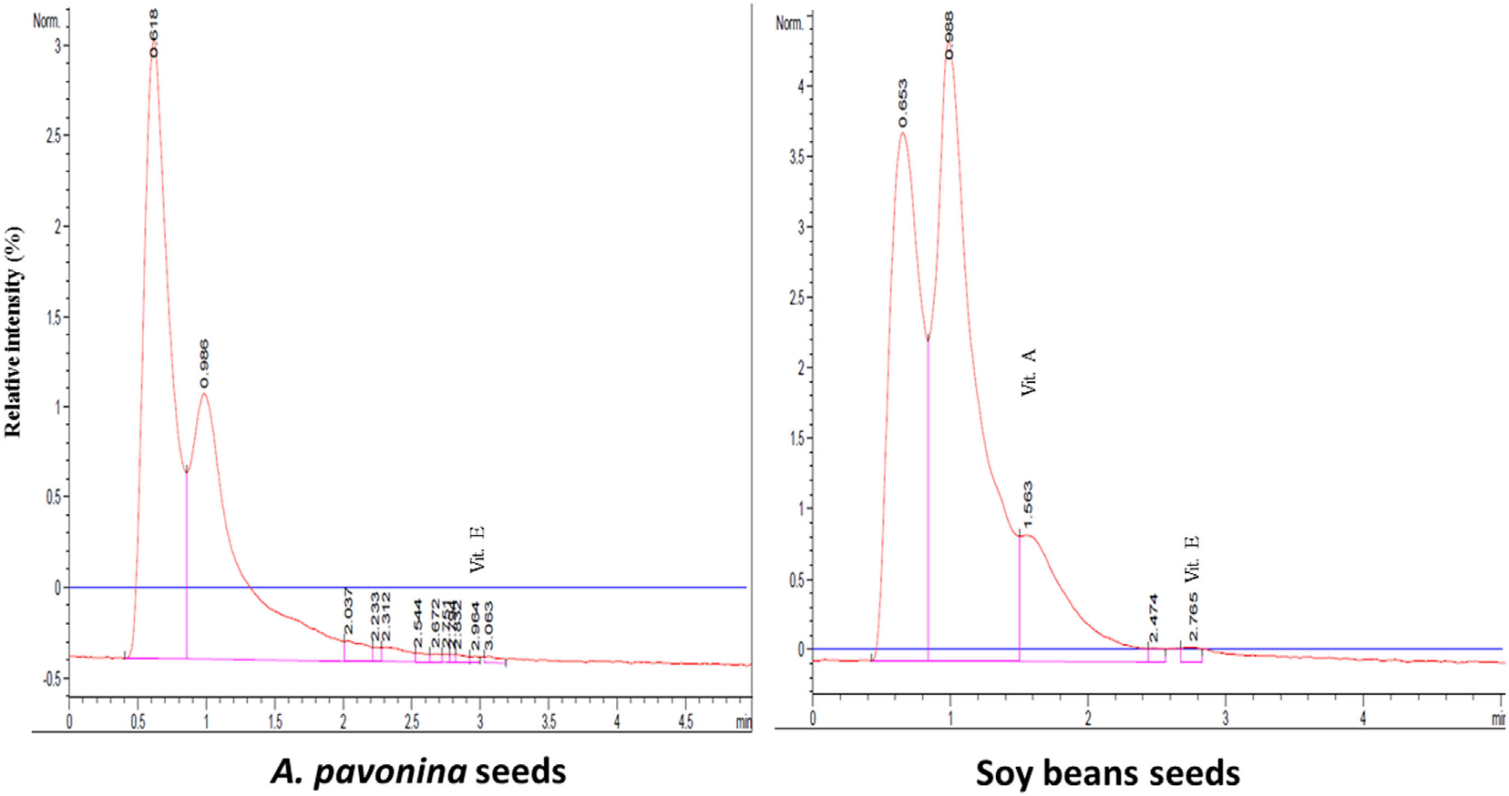
Chromatogram for the three fat-soluble vitamins in pro-milk extracted from *A. pavonina* and soy bean.

**Figure 2:**
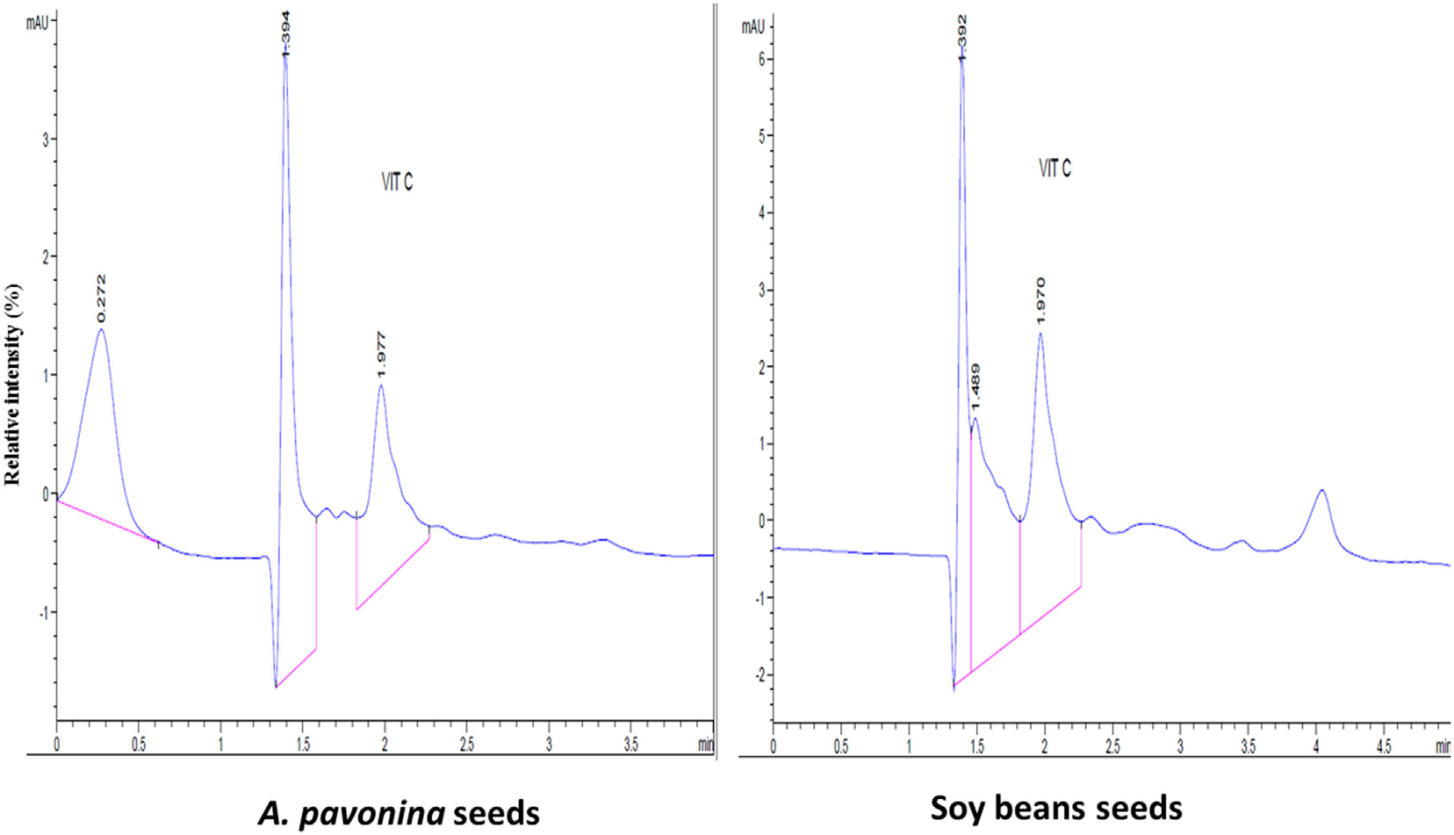
Chromatogram for vitamin C in pro-milk extracted from *A. pavonina* and soy bean.

**Figure 3:**
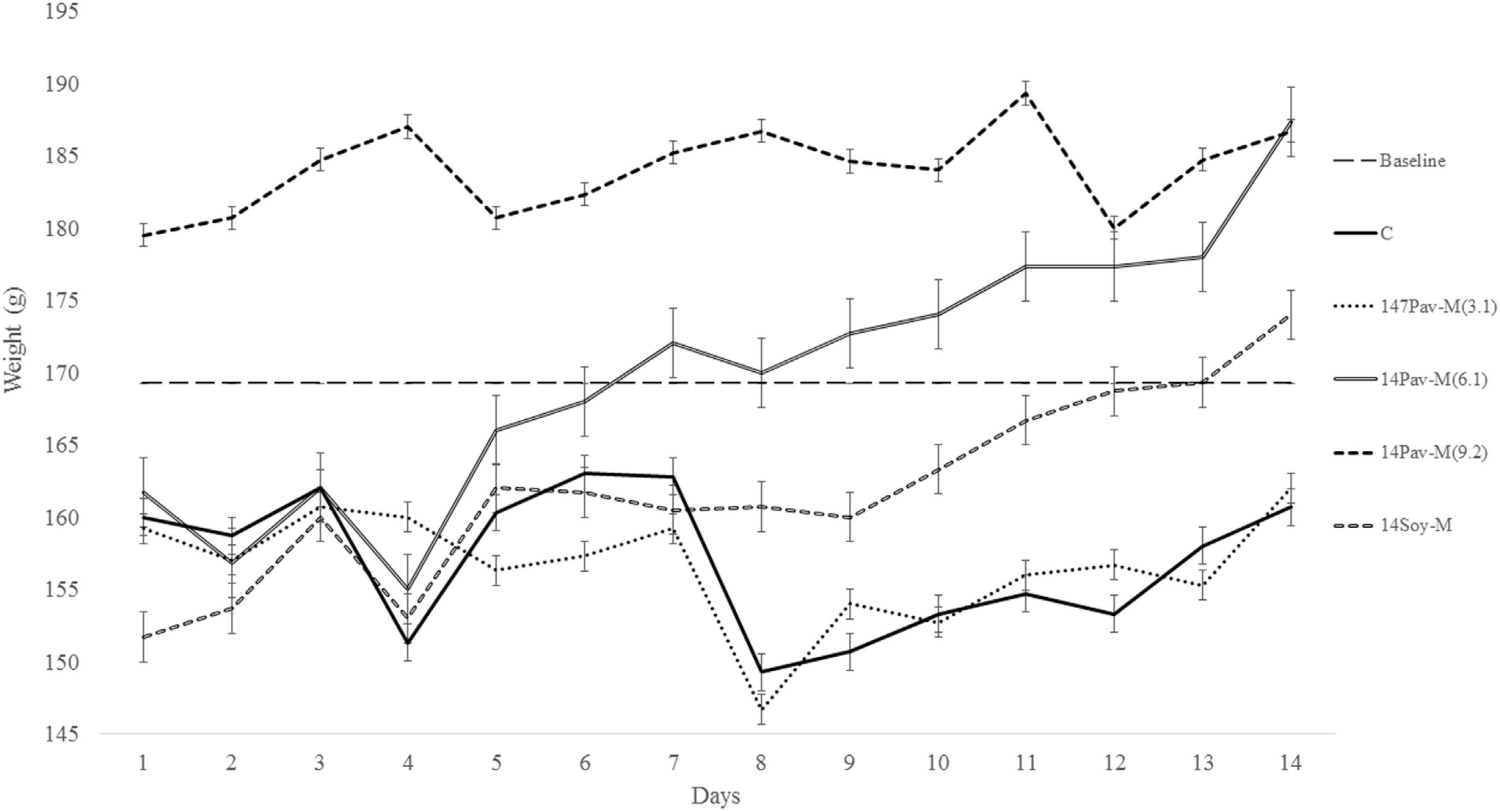
Changes in body weight during administration of different dosage of milk extracted from *A. pavonina* and soy bean. C, control; 14Pav-M(3.1), group with daily administration of 3.1 µl/g animal wt. pro-milk extract from *A. pavonina* seed; 14Pav-M(6.1), group with daily administration of 6.1 µl/g animal wt. pro-milk extract from *A. pavonina* seed; 14Pav-M(9.2), group with daily administration of 9.2 µl/g animal wt. pro-milk extract from *A. pavonina* seed; 14Soy-M, group with daily administration of 6.1 µl/g animal wt. pro-milk extract from soy bean seed.

The resultant chromatograms for the standard vitamins are indicated in Figures S1–S4 in Supplementary material, while those of the vitamins detected in the pro-milk extract from *A. pavonina* seeds and soy seeds are indicated in Figures 1–3. The pro-milk extract from *A. pavonina* seeds was significantly higher (*P* < 0.05) in protein, Mg, K, Na, and Cu but significantly lower (*P* < 0.05) in Ca, Mn, vitamin A, C, and E compared to those of Soy-M (Table 2).

**Table 2:** Some basic nutritional profile of milk extract from *Adenanthera pavonina* and soy bean.

Minerals	Concentration				
	A Pav-M	Soy-M	Silk soy milk^†^	Commercial soy milk^†^	Recommended daily intake^†^
Protein (%)	36.14 ± 0.12^a^	5.12 ± 0.06^b^	X	30.08 (g/l)	0.8 g/kg body weight
Ca (mg/l)	440.99 ± 0.93^a^	594.96 ± 1.06^b^	1,265.8	1,404.2	1,000 mg
Mg (mg/l)	96.69 ± 0.03^a^	76.79 ± 1.13^b^	X	X	400 mg
K (mg/l)	190.41 ± 0.11^a^	46.99 ± 0.00^b^	1,265.8	475.0	3,500 mg
Na (mg/l)	64.24 ± 0.24^a^	60.59 ± 0.16^b^	358.7	916.7	2,400 mg
Zn (mg/l)	0.67 ± 0.75	0.66 ± 0.75	X	X	15 mg
Cu (mg/l)	0.55 ± 0.24^a^	0.09 ± 0.01^b^	X	X	2 mg
Fe (mg/l)	0.21 ± 0.14	0.18 ± 0.01	X	4.17	18 mg
Cd (mg/l)	0.01 ± 0.01	0.02 ± 0.00	X	X	3.6 μg/kg bw
Mn (mg/l)	0.04 ± 0.01^a^	0.07 ± 0.00^b^	X	X	2 mg
Cr (mg/l)	0.07 ± 0.03	0.05 ± 0.00	X	X	0.20 mg
Pb (mg/l)	ND	ND	X	X	1.0 μg/kg bw
Vitamin A (mg/100 g)	ND^a^	24.2 ± 0.31^b^	2,109.7 (I.U.)	95.8 (I.U.)	5,000 (I.U.)
Vitamin C (mg/100 g)	1.87 ± 0.01^a^	4.65 ± 0.07^b^	0	0.83 mg/l	60 mg
Vitamin D (mg/100 g)	ND	ND	506.3 (I.U.)	0.83 mg/l	400 (I.U.)
Vitamin E (mg/100 g)	0.12 ± 0.01^a^	0.70 ± 0.00^b^	X	X	30 (I.U.)

*ND, not detected; X, not declared; I.U., International unit*.

*Value = mean ± SD (n = 2)*.

*Data within each row with different superscript alphabets are statistically different (P < 0.05)*.

*^†^Sources (13, 41–45)*.

### Changes in Weight during Administration of the Pro-Milk Extracts

The groups administered the highest dosage (1.5 ml) of pro-milk extract from *A. pavonina* was increased significantly (*P* < 0.05) in body weight throughout the first 13-day period (Figure 3). The administration of the high dosage (1.5 ml) of pro-milk extract from *A. pavonina* significantly increased (*P* < 0.05) the weight of the kidney within 7 days. The weight of the small intestine was also significantly increased (*P* < 0.05) by the pro-milk extract from *A. pavonina* (1.0–1.5 ml) and the soy milk extract (Table 3).

**Table 3:** Changes in weight of organs of rats administered with pro-milk extract from *Adenanthera pavonina* and soy bean.

Parameters	Brain		Heart		Liver		Kidney		Small intestine	
	Day 7	Day 14	Day 7	Day 14	Day 7	Day 14	Day 7	Day 14	Day 7	Day 14
Baseline	1.43 ± 0.06	1.43 ± 0.06	0.63 ± 0.06	0.63 ± 0.06	6.70 ± 0.99	6.70 ± 0.99	1.17 ± 0.21	1.17 ± 0.21	7.43 ± 2.36^a^	7.43 ± 2.36
C	1.37 ± 0.06	1.27 ± 0.06	0.60 ± 0.00	0.57 ± 0.06	6.73 ± 0.29	5.53 ± 0.31	1.00 ± 0.00^a^	0.90 ± 0.10	4.70 ± 0.72^b^	8.97 ± 1.72
Pav-M(3.1)	1.17 ± 0.23	1.27 ± 0.06	0.6 ± 0.10	0.53 ± 0.06	6.30 ± 0.27	5.63 ± 0.59	1.03 ± 0.06	0.97 ± 0.06	6.57 ± 0.93	7.83 ± 0.59
Pav-M(6.1)	1.27 ± 0.15	1.40 ± 0.17	0.53 ± 0.06	0.67 ± 0.15	6.70 ± 0.35	6.47 ± 0.31	1.00 ± 0.00	1.00 ± 0.10	8.17 ± 0.71^c^	8.20 ± 1.30
Pav-M(9.2)	1.57 ± 0.15	1.47 ± 0.06	0.6 ± 0.00	0.57 ± 0.06	7.10 ± 1.08	6.03 ± 0.25	1.30 ± 0.17^b^	1.07 ± 0.12	8.67 ± 2.58^d^	9.40 ± 1.00
Soy-M	1.40 ± 0.10	1.33 ± 0.06	0.57 ± 0.06	0.57 ± 0.06	6.60 ± 0.36	6.30 ± 0.00	1.03 ± 0.06	1.03 ± 0.06	9.33 ± 0.76^d^	10.60 ± 0.62

*Value = mean ± SD (n = 3)*.

*Data within each column with different superscript alphabets are statistically different (P < 0.05)*.

*C, control; Pav-M(3.1), group with daily administration of 3.1 µl/g animal wt. pro-milk extract from *A. pavonina* seed; Pav-M(6.1), group with daily administration of 6.1 µl/g animal wt. pro-milk extract from A. pavonina seed; Pav-M(9.2), group with daily administration of 9.2 µl/g animal wt. pro-milk extract from A. pavonina seed; Soy-M, group with daily administration of 6.1 µl/g animal wt. pro-milk extract from soy bean seed*.

*7 or 14 is added before each abbreviation within text to indicate the days of administration*.

### Total Protein of Organs

Both, the 7Pav-M(6.1) and 7Pav-M(9.2) groups showed a significant increase (*P* < 0.05) in the protein level in the heart (Table 4). The groups, 7Pav-M(3.1) and 7Soy-M at day 7, and 14Soy-M and 14Pav-M(6.1) at the second week showed a significant reduction (*P* < 0.05) in the protein level in the liver, whereas 7Pav-M(9.2) showed a significant increase in the protein level in the liver at day 7 (Table 4).

**Table 4:** Total protein levels in organs of rats administered with pro-milk extract from *Adenanthera pavonina* and soy bean.

Parameters	Brain		Heart		Liver		Kidney		Small intestine	
	Day 7	Day 14	Day 7	Day 14	Day 7	Day 14	Day 7	Day 14	Day 7	Day 14
Baseline	8.67 ± 1.41	8.67 ± 1.41	8.51 ± 0.54^b^	8.51 ± 0.54^b^	8.28 ± 0.16	8.28 ± 0.16	8.53 ± 0.35^b^	8.53 ± 0.35^b^	7.90 ± 0.05	7.90 ± 0.05
C	7.64 ± 0.23	7.18 ± 0.01	6.76 ± 0.20^a^	7.16 ± 0.00^a^	8.53 ± 0.00^a^	8.42 ± 0.01^a^	8.22 ± 0.04^a^	8.03 ± 0.00^a^	7.98 ± 0.07^a^	7.92 ± 0.01^a^
Pav-M(3.1)	9.27 ± 0.51	9.50 ± 0.06	7.56 ± 0.29	7.49 ± 0.06	8.09 ± 0.09^b^	8.11 ± 0.01	8.65 ± 0.05^b^	8.91 ± 0.13^b^	8.51 ± 0.30^b^	8.75 ± 0.03^b^
Pav-M(6.1)	6.86 ± 0.01	7.74 ± 0.27	8.15 ± 1.12^b^	7.30 ± 0.40	8.24 ± 0.18	7.87 ± 0.2^b^	8.18 ± 0.01	8.52 ± 0.03^b^	7.47 ± 0.00^b^	8.10 ± 0.19^b^
Pav-M(9.2)	7.48 ± 0.00	8.58 ± 1.09	7.81 ± 0.02^b^	7.58 ± 0.32	8.59 ± 0.07	8.28 ± 0.23	8.48 ± 0.05	8.80 ± 0.03^b^	8.20 ± 0.04^b^	8.28 ± 0.04^b^
Soy-M	8.66 ± 0.16	7.98 ± 1.34	7.23 ± 0.05	7.25 ± 0.31	7.58 ± 0.0^c^	7.78 ± 0.32^b^	8.79 ± 0.02^b^	8.45 ± 0.11^b^	8.57 ± 0.09^b^	8.37 ± 0.33^b^

*Value = mean ± SD (n = 3)*.

*Data within each column with different superscript alphabets are statistically different (P < 0.05)*.

*C, control; Pav-M(3.1), group with daily administration of 3.1 µl/g animal wt. pro-milk extract from A. pavonina seed; Pav-M(6.1), group with daily administration of 6.1 µl/g animal wt. pro-milk extract from A. pavonina seed; Pav-M(9.2), group with daily administration of 9.2 µl/g animal wt. pro-milk extract from A. pavonina seed; Soy-M, group with daily administration of 6.1 µl/g animal wt. pro-milk extract from soy bean seed*.

*7 or 14 is added before each abbreviation within text to indicate the days of administration*.

The 7Pav-M(3.1) and 7Soy-M groups and all the groups administered all the three volumes of pro-milk extracts from *A. pavonina* seed and soy bean seed showed a significant increase (*P* < 0.05) in the protein level in the kidney on day 14 (Table 4). On day 7, the 7Pav-M(3.1) and 7Soy-M groups, and on day 14, the 14Pav-M(9.2), 14Soy-M, and 14Pav-M(3.1) groups showed a significant increase (*P* < 0.05), while the 7Pav-M(6.1) group showed a significant decrease, in the level of protein in the small intestine within 7-day period (Table 4).

### Cytochrome P450 Activity

The groups, 7Soy-M by day 7, and 14Pav-M(9.2), 14Soy-M, 14Pav-M(6.1), and 14Pav-M(3.1) by day 14 showed a significant reduction (*P* < 0.05) in cytochrome P450 activity in the brain (Table 5). The groups, 7Pav-M(9.2) and 7Pav-M(6.1) showed a significant reduction (*P* < 0.05) in cytochrome P450 activity in the heart by day 7 (Table 5). Also, the 7Pav-M(6.1) showed a significant reduction (*P* < 0.05) in cytochrome P450 activity in the liver by day 7, but showed a significant increase (*P* < 0.05) in cytochrome P450 activity in the kidney (Table 5). The daily administration of all the pro-milk extracts from *A. pavonina* [7Pav-M(3.1–9.2)] by day 7, and that from both soy beans and *A. pavonina* [14Soy-M and 14Pav-M(6.1)] by day 14 showed a significant increase (*P* < 0.05) in the cytochrome P450 activity in the intestine (Table 5).

**Table 5:** Cytochrome P450 activities in organs of rats administered with milk extract from *Adenanthera pavonina* and soy bean.

Parameters	Brain		Heart		Liver		Kidney		Small intestine	
	Day 7	Day 14	Day 7	Day 14	Day 7	Day 14	Day 7	Day 14	Day 7	Day 14
Baseline	1.06 ± 0.60^b^	1.06 ± 0.60^b^	0.73 ± 0.16	0.73 ± 0.16	0.64 ± 0.37	0.64 ± 0.37	0.67 ± 0.10	0.67 ± 0.10	2.13 ± 0.13^b^	2.13 ± 0.13^b^
C	1.86 ± 0.04^a^	2.18 ± 0.09^a^	1.17 ± 0.02^a^	1.04 ± 0.01^a^	0.73 ± 0.43^a^	0.77 ± 0.18	0.61 ± 0.39^a^	0.61 ± 0.02^a^	0.65 ± 0.28^a^	0.60 ± 0.03^a^
Pav-M(3.1)	1.27 ± 0.46	1.34 ± 0.04^b^	1.27 ± 0.60	1.07 ± 0.12	0.44 ± 0.00	0.76 ± 0.13	0.24 ± 0.25	0.55 ± 0.02	1.45 ± 0.07^b^	0.58 ± 0.02
Pav-M(6.1)	1.90 ± 0.13	0.99 ± 0.05^b^	0.51 ± 0.02^b^	0.90 ± 0.19^b^	0.06 ± 0.08^b^	0.75 ± 0.02	1.53 ± 0.46^b^	0.23 ± 0.30^b^	1.12 ± 0.26^b^	1.70 ± 0.24^b^
Pav-M(9.2)	1.32 ± 0.09	0.82 ± 0.10^b^	0.38 ± 0.02^b^	1.14 ± 0.11^b^	0.20 ± 0.05	0.37 ± 0.09	0.47 ± 0.35	0.42 ± 0.05	1.37 ± 0.21^b^	0.34 ± 0.27
Soy-M	0.72 ± 0.03^b^	0.84 ± 0.44^b^	0.10 ± 0.04	1.14 ± 0.40	1.16 ± 0.31	0.58 ± 0.31	0.62 ± 0.06	0.42 ± 0.01	0.27 ± 0.12	1.17 ± 0.04^b^

*Value = mean ± SD (n = 3)*.

*Data within each column with different superscript alphabets are statistically different (P < 0.05)*.

*C, control; Pav-M(3.1), group with daily administration of 3.1 µl/g animal wt. pro-milk extract from A. pavonina seed; Pav-M(6.1), group with daily administration of 6.1 µl/g animal wt. pro-milk extract from A. pavonina seed; Pav-M(9.2), group with daily administration of 9.2 µl/g animal wt. pro-milk extract from A. pavonina seed; Soy-M, group with daily administration of 6.1 µl/g animal wt. pro-milk extract from soy bean seed*.

*7 or 14 is added before each abbreviation within text to indicate the days of administration*.

### Acetylcholinesterase Activity

The acetylcholinesterase activity in the liver was significantly reduced (*P* < 0.05) in the 14Pav-M(6.1) and 14Pav-M(3.1) groups on days 7 and 14, respectively. The acetylcholinesterase activity was significantly decreased (*P* < 0.05) on day 14 in the intestine of rats in the 14Pav-M(9.2) group, in the heart and kidney of rats in the 14Pav-M(3.1–9.2) group, and in the liver and kidney of rats in the 14Soy-M group (Table 6). Acetylcholinesterase activity was significantly increased (*P* < 0.05) in the liver of the rats in the 14Pav-M(6.1) group on day 14, and in the 14Soy-M group on day 7 (Table 6).

**Table 6:** Acetylcholinesterase activities in organs of rats administered with milk extract from *Adenanthera pavonina* and soy bean.

Parameters	Brain		Heart		Liver		Kidney		Small intestine	
	Day 7	Day 14	Day 7	Day 14	Day 7	Day 14	Day 7	Day 14	Day 7	Day 14
Baseline	2.41 ± 1.21	2.41 ± 1.21	0.82 ± 0.24	0.82 ± 0.24^b^	0.45 ± 0.03	0.45 ± 0.03	0.76 ± 0.00^b^	0.76 ± 0.00^b^	0.87 ± 0.43	0.87 ± 0.43
C	1.23 ± 0.02	1.24 ± 0.02	1.21 ± 0.14	1.74 ± 0.10^a^	0.53 ± 0.17^a^	0.59 ± 0.17^a^	0.35 ± 0.19^a^	1.10 ± 0.11^a^	0.45 ± 0.42	1.04 ± 0.13^a^
Pav-M(3.1)	1.36 ± 0.67	0.72 ± 0.02	1.63 ± 1.40	0.70 ± 0.05^b^	0.25 ± 0.17	0.21 ± 0.00^b^	0.61 ± 0.08	0.74 ± 0.09^b^	0.88 ± 0.25	0.74 ± 0.08
Pav-M(6.1)	1.13 ± 0.05	1.28 ± 0.57	0.56 ± 0.02	1.10 ± 0.10^b^	0.48 ± 0.01^b^	0.95 ± 0.13^b^	0.62 ± 0.07	0.68 ± 0.22^b^	0.55 ± 0.04	0.86 ± 0.17
Pav-M(9.2)	1.03 ± 0.04	1.08 ± 0.21	0.30 ± 0.07	1.95 ± 0.14^b^	0.29 ± 0.26	0.26 ± 0.36	0.22 ± 0.02	0.51 ± 0.08^b^	0.25 ± 0.01	0.33 ± 0.17^b^
Soy-M	0.54 ± 0.70	2.34 ± 0.20	1.06 ± 0.32	0.93 ± 0.05	0.69 ± 0.02^b^	0.16 ± 0.09^b^	0.45 ± 0.03	0.22 ± 0.23^b^	0.15 ± 0.12	0.94 ± 0.36

*Value = mean ± SD (n = 3)*.

*Data within each column with different superscript alphabets are statistically different (P < 0.05)*.

*C, control; Pav-M(3.1), group with daily administration of 3.1 µl/g animal wt. pro-milk extract from A. pavonina seed; Pav-M(6.1), group with daily administration of 6.1 µl/g animal wt. pro-milk extract from A. pavonina seed; Pav-M(9.2), group with daily administration of 9.2 µl/g animal wt. pro-milk extract from A. pavonina seed; Soy-M, group with daily administration of 6.1 µl/g animal wt. pro-milk extract from soy bean seed*.

*7 or 14 is added before each abbreviation within text to indicate the days of administration*.

### ATPases Activity

The 14Pav-M(3.1) group on day 14 showed a significant reduction (*P* < 0.05) in the Ca^2+^-ATPase activity (Table 7), while the 14Pav-M(9.2) group showed a significant increase (*P* < 0.05) in Mg^2+^-ATPase activity (Table 7) in erythrocyte membrane.

**Table 7:** Adenosine triphosphate synthase (ATPase) activities in blood of rats administered with milk extract from *Adenanthera pavonina* and soy bean.

Parameters	Na^+^-K^+^ ATPase (M/ml blood sample)		Ca^2+^ ATPase (M/ml blood sample)		Mg^2+^ ATPase (M/ml blood sample)	
	Day 7	Day 14	Day 7	Day 14	Day 7	Day 14
Baseline	9.02 ± 1.01	9.02 ± 1.01	6.70 ± 1.28	6.70 ± 1.28^b^	6.05 ± 3.52^b^	6.05 ± 3.52^b^
C	6.54 ± 0.50	13.13 ± 1.49	9.41 ± 2.91	14.50 ± 0.46^a^	10.45 ± 3.14^a^	17.91 ± 0.49^a^
Pav-M(3.1)	6.31 ± 2.50	14.67 ± 0.92	7.83 ± 3.28	8.29 ± 9.77^b^	9.40 ± 3.33	17.55 ± 1.06
Pav-M(6.1)	6.69 ± 4.05	12.47 ± 1.79	9.63 ± 0.37	13.68 ± 1.23	10.06 ± 1.36	17.52 ± 0.61
Pav-M(9.2)	5.77 ± 0.15	13.10 ± 1.24	8.22 ± 1.03	14.81 ± 0.47	8.84 ± 0.87	22.96 ± 4.53^b^
Soy-M	7.39 ± 0.84	12.34 ± 1.61	8.38 ± 1.30	13.64 ± 2.51	12.02 ± 0.33	18.35 ± 3.96

*Value = mean ± SD (n = 3)*.

*Data within each column with different superscript alphabets are statistically different (P < 0.05)*.

*C, control; Pav-M(3.1), group with daily administration of 3.1 µl/g animal wt. pro-milk extract from A. pavonina seed; Pav-M(6.1), group with daily administration of 6.1 µl/g animal wt. pro-milk extract from A. pavonina seed; Pav-M(9.2), group with daily administration of 9.2 µl/g animal wt. pro-milk extract from A. pavonina seed; Soy-M, group with daily administration of 6.1 µl/g animal wt. pro-milk extract from soy bean seed*.

*7 or 14 is added before each abbreviation within text to indicate the days of administration*.

## Discussion

Milk is considered to be a vital food for humans as it contains approximately all different essential nutrients (46). Consumers expect that protein, minerals (especially calcium), and vitamins will be provided by milk and milk products. The presence of these nutrients is therefore an indicator of any dairy food products to be suitable for consumption. In this study, we examined the physicochemical, nutritional, and biochemical qualities of a pro-milk extract from *A. pavonina* seed to serve as a milk for human and livestock consumption.

### Physicochemical Properties of the Pro-Milk Extracts

The two pro-milk extracts have a similar density (Table 1), suggesting the possibility of also bottling the pro-milk from *A. pavonina* for commercialization and industrial purposes. Manufacturing industries are directing their efforts at producing dairy products of vegetable origin (10, 47). Research efforts on producing milk from soy beans has been successfully been transformed to industrial production and commercialization by companies such as Greenspot (Thailand) Co producing Vitamilk and CHI Nigeria limited, Nigeria producing Chi Soya Milk. Color is usually the first parameter that consumers use to judge the quality of milk (48). The pattern by which L*, a*, and b* color indices represents how humans appreciates different color has been described (35). L*, a*, and b* values of commercial milks earlier reported ranges of 81.7–88.1, (−4.8)–(−0.2), and 4.1–9.1, respectively (48, 49). The extent of lightness for both the pro-milk extracted from *A. pavonina* and soy beans were lower than this range (Table 1), although that of the extract from soy beans was closer to the value. It is likely that plant milk could generally be less bright than their corresponding animal based or synthesized milk products. Also, the pro-milk extract from *A. pavonina* was more reddish in color than that from soy beans, which has its green color to be closely lower than those of earlier reported commercial animal milk. Both pro-milk extracts from *A. pavonina* and soy bean seeds have their yellow color values within the range of the commercial milk. Considering the hue values and CI, of the pro-milk extracts, that provides how human perceives the milk-like extract (50), it was observed that both extracts are slightly different as indicated by the value (15.33 ± 1.67) of the color difference between the two extracts (Table 1).

### Nutritional Qualities of Pro-Milk Extract from *A. pavonina*

The pro-milk extract from A Pavonina contains higher amount protein, Mg, K, Na, and Cu compared to that from soy bean. It also contains lower amount of Ca, Mn, vitamin A, C, and E compared to those of Soy-M (Table 2). Although the amount of these element (Ca and manganese) may be sufficient to sustain human’s health (28), it may be essential to boost the levels of the vitamins for the pro-milk meet the daily requirement of each vitamins for humans. The pro-milk extract from *A. pavonina* seeds (APav-M) and soy bean (Soy-M) contained a comparable amount of Zn, Fe, Cd, Cr, and Pb. The level of the vitamin D and Pb were too low to be detected in both extract. Na, Fe, Zn, Cu, and K in addition to P and Al that were not examined in this study had been similarly reported to be in sufficient amount in oil extracted from the *A. pavonina* seed (51).

The levels of vitamins and some other nutrients (protein—3.4–3.5 mg/g, calcium—0.2 mg/g, vitamin C—8.53–9.82 mg/100 g, and vitamin E—0.2 mg/100 g) detected in the pro-milk extracts from soy bean (Table 2) were comparable to those of other earlier reports (52–55). Also, the undetectable level of vitamin D in the pro-milk extract from the soy bean further support earlier report of vitamin D deficiency among infants fed on soy milk (56). These also validate the nutritional status of the pro-milk extracts from *A. pavonina* seeds (Table 2). Further, condensation or concentration of the pro-milk extracts with the aid of appropriate technique (such as freeze-drying) during production may be required to maximize the health benefits to consumers and to meet up with the amount declared by the previously fortified commercialized plant milk indicated in Table 2 (57–61).

### Changes in Weight Due to the Oral Administration of Extracts

Feeding on 9.2 µl/g of pro-milk extract from *A. pavonina* generally resulted in better growth (Figure 3). This growth may be due to the provision of sufficient amount of protein and some minerals by the high intake of the *A. pavonina* pro-milk extract (Table 2), the pleasant aroma from the pro-milk that was noted during the experimentation. The daily administration of the 1.5 ml and 1.0–1.5 ml pro-milk extract from *A. pavonina* resulted in the early growth in the kidney and the small intestine, respectively, at day 7 (Table 3). Soy-M was only able to induce a similar growth in the small intestine. The metabolic effect of nutrient intake is rapidly manifested in the intestine that first interacts with such a nutrient, and later in the kidney. In this case, the increased weight for these two organs may be largely due to the rich nutrients including protein (Table 2) in the pro-milk extract from the *A. pavonina* seed (62, 63).

It was previously established that toxicity and stress induces loss in weight (64, 65). Methanol extract of the *A. pavonina* seeds was reported to have hepatoprotective properties in previous acute toxicity and histopathological studies (66). The methanol extract from the *A. pavonina* seeds was reported to induce 24 h motality between 100 and 3,200 mg/kg when intraperitoneally administered. Although, there was no motality reported in another study at 2,000 mg/kg, which established the LD50 of the methanol extract of *A. pavonina* seeds to be 1,360 mg/kg (67, 68). It is worthwhile to note that there was no mortality observed in this present study despite the higher concentration (3.03–9.09 g/kg, as extrapolated from the 1.01 g/ml density of the pro-milk extract) of the pro-milk from *A. pavonina* seeds administered. Instead, the pro-milk extract provoked a continuous increase in weight (Figure 3). Similar increase in weight due to the 30 days intraperitoneal administration of methanolic extract from *A. pavonina* was previously reported (31). The favorable growth may be due to reduction or detoxification of potential toxic compound(s) in the seeds by the combined microwaving and the 48 h soaking induced fermentation process during the production of the pro-milk extracts (1, 69, 70). It is further suggested that LD50 and the histopathological studies be carried out on the pro-milk extract for *A. pavonina* seed.

### The Effect of Administration of the Pro-Milk Extracts on Protein and Some Biochemical Indices

Most toxic substances will in most cases lead to either the damaging of the nervous system or disrupt the membrane integrity of some organs in the body (65, 71–74). Acetylcholine esterase, cytochrome P450, and the ATPases were selected as biochemical markers for monitoring the toxicity of the pro-milk extracts as they are key enzymes that are responsible for the destruction of nervous system and leakages during damage to membrane integrity in vital organs of the rats.

All the different dosages of the extract did not have any effect at all on the total protein and the acetylcholinesterase activity in the brain (Tables 4, 6 and 7). All the dosages of *A. pavonina* pro-milk extracts were found to increase the level of total protein in all the organs (with the exception of the brain) both in the first and second week (Table 4). Increase in the total protein in these organs indicated the healthy growth of the whole body and further confirms that the pro-milk extract from *A. pavonina* is nutritionally rich to support growth (75).

### Cytochrome P450

Cytochrome P450 is one of the enzymes involved in biotransformation of xenobiotics and various hydrophobic endogenous compounds such as vitamins, bile acids, and fatty acids, into their hydrophilic forms (76). Metabolism of drugs by microsomal cytochrome P450 plays an important role in the pharmacological and toxicological effects of drugs and in the drug–drug and food–drug interactions in humans (77, 78). Diet modulates cytochrome P450 to determine the activity of drugs. A good diet will stimulate the activity of these enzymes to clear harmful xenobiotics and other chemicals from the body resulting in a healthy living (77, 78).

Cytochrome P450 activity in the kidney was increased by the daily administration of 6.1 µl/g pro-milk extract from *A. pavonina* within 7 days, and in the intestine at all dosages (3.1–9.2 µl/g) within 7 days, which was sustained at a dosage of 6.1 µl/g for upto 14 days (Table 5). These findings indicate that the consumption of the pro-milk extract may enhance the clearance of xenobiotics and drugs at appropriate dosage in both the kidney and the intestine (77, 78). The daily administration of the pro-milk extracts from *A. pavonina* (6.1–9.2 µl/g) required 7 and 14 days to reduce cytochrome P450 activity in both, the liver and the heart, and in the brain, respectively (Table 5). This indicates that the ability of cytochrome P450 to clear xenobiotics from the liver, heart, and the brain may be impaired above the 0.5 ml dosage when administered daily (77, 78).

### Acetylcholinesterase

Acetylcholinesterase also known as acetylhydrolase terminates the neuro-transmitting effect of acetylcholine by hydrolyzing the compound to acetyl-coA and choline (79). Acetylcholine plays an important role in memory, thinking, and behavioral and psychological activities in humans (79). A decline in this neurotransmitter due to the increase in acetylcholinesterase can lead to diseases associated with neuronal dysfunctions. The activity of acetylcholinesterase was reduced in the liver, intestine, heart, and kidney by the daily administration of the pro-milk extracts from *A. pavonina* seeds for 14 days. The reduction of the acetylcholinesterase activity in both the liver and kidney due to the daily administration of pro-milk extract from soy bean seed for 14 days was also observed (Table 6). Thus, the pro-milk extract from *A. pavonina* acts as an acetylcholinesterase inhibitor, and therefore it could be useful as a drug for preventing Alzheimer’s disease development by reducing or stopping acetylcholine breakdown (80). Only the daily administration of 6.1 µl/g of the pro-milk extract from *A. pavonina* seed and soy bean seed increased acetylcholine activity in the liver within 14 and 7 days, respectively (Table 6), indicating the possibility of the two pro-milk extract of inducing a neurological side effect in the liver. Finally, the activity of the acetylcholinesterase in the brain was normal in response to the administration of all the dosage of the pro-milk extract from *A. pavonina* seed and that from soy bean seed (Table 6). The biomolecule(s) modulating the acetylcholinesterase activity could have been prevented from entering the brain by the blood–brain barrier (79). This encouraging effect of the pro-milk extract from *A. pavonina* on the integrity of the neurological system further confirmed the low heavy metal content in the pro-milk reported in this study (Table 2).

### Adenosine Triphosphate Synthase

The Ca^2+^-, Mg^2+^-, and Na^+^-K^+^-ATPase are responsible for the movement of calcium, magnesium, and sodium ions, respectively. Nutrients and other chemicals across the cell membrane are vital to the process of generating energy and its utilization for driving other vital processes required for maintenance of health (81). Physiologically, Na^+^-K^+^-ATPase present in organs such as the intestines and the kidney regulates fluid reabsorption and electrolyte movement by establishing an ionic gradient across epithelial membranes. It is estimated that approximately 23% of the ATP utilized in humans at rest is utilized by the sodium pump (82). Na^+^-K^+^-ATPase is unique in that it is specifically inhibited by cardiac glycosides such as ouabain and digoxin (81). The administration of the pro-milk extracts from *A. pavonina* and soy bean preserved the integrity of the membrane in the erythrocyte with respect to the transportation of Na^+^ and K^+^, as the activity of Na^+^-K^+^-ATPase was not affected (Table 7). The daily consumption of the pro-milk extract from *A. pavonina* at low dose (3.1 µl/g) for 14 days impeded the activity of Ca^2+^-ATPase while the consumption at high dose (9.2 µl/g) for 14 days facilitated the activity of Mg^2+^-ATPase. Also, we may attribute the depleted state of the Ca^2+^-ATPase on day 14 of consumption to the low bioavailability of the calcium in the pro-milk extracts from *A. pavonina* seed (Table 2). Thus, it may be necessary to consume the pro-milk extract either at higher dosage (9.2 µl/g) that supported the highest weight gain (Figure 3), or for a longer period than 14 days to supply more magnesium ion to overcome the impaired function of the calcium ion-dependent ATPase.

In conclusion, *A. pavonina* seed extract is richer in protein and minerals but lower vitamins than extracts from soy beans seeds. It may therefore provide nutritional benefits to human and livestocks. The pro-milk extract from *A. pavonina* seed and that from soy bean seed may complement each other to provide health and nutritional benefits. Further extensive studies on the pharmacological and toxicological effects of the pro-milk extracts are recommended.

## Ethics Statement

An ethical permit (reference number CU/BIOSCRECU/BIO/2015/010) was obtained from the ethics committee of our institution prior to beginning the study. Handling of the experimental rats was in accordance with the ethical procedures recommended by the Covenant University Ethical Committee.

## Author Contributions

IA discovered the potential of the plant for generating milk, designed the study, performed the statistical analysis, determined the color parameters, interpreted the data, and prepared the manuscript. IN and OM performed the mineral analysis. CE, RW, OA, and TO treated the experimental rats, sacrificed them, performed the biochemical analysis, and interpreted the data.

## Conflict of Interest Statement

The authors declare that the research was conducted in the absence of any commercial or financial relationships that could be construed as a potential conflict of interest.
