# Supplementary figures and images for: Production of a New Plant-Based Milk from Adenanthera pavonina Seed and Evaluation of Its Nutritional and Health Benefits
Source: Front Nutr. 2018 Feb 12;5:9. doi: 10.3389/fnut.2018.00009 (PMC5845130; doi:10.3389/fnut.2018.00009)

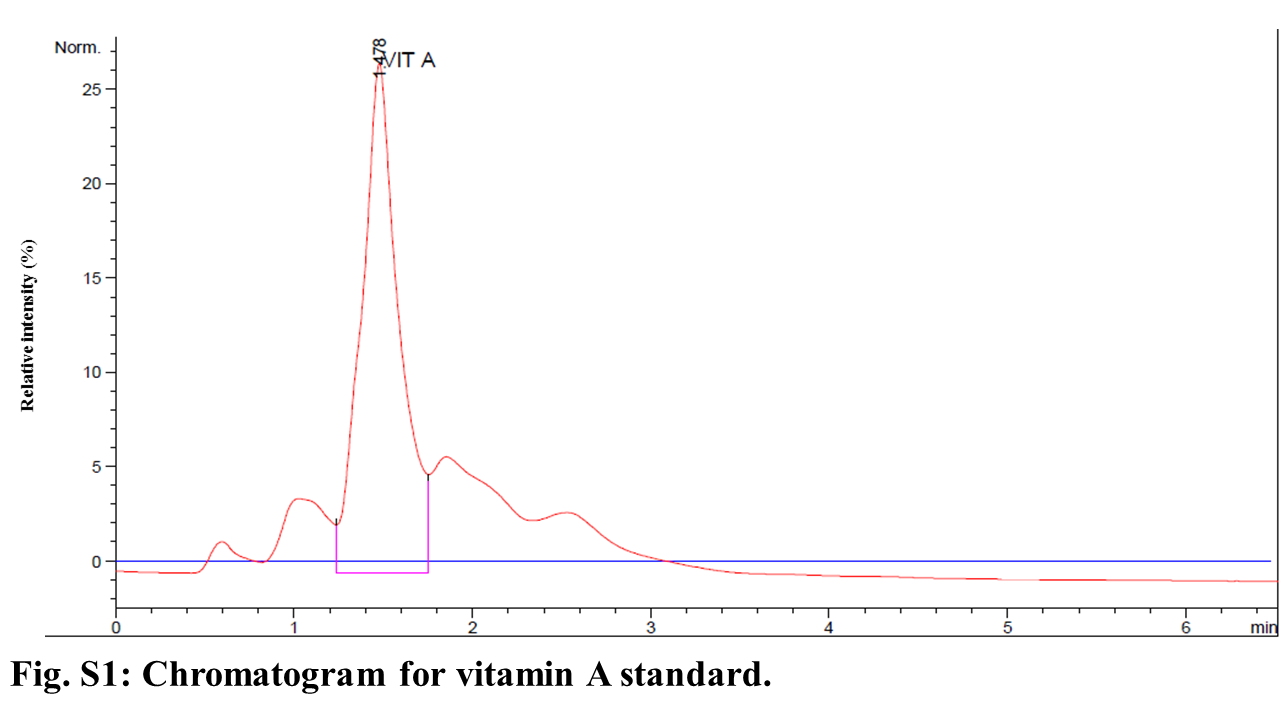

Supplement: Supplementary file 1 [file Image_1.tif]

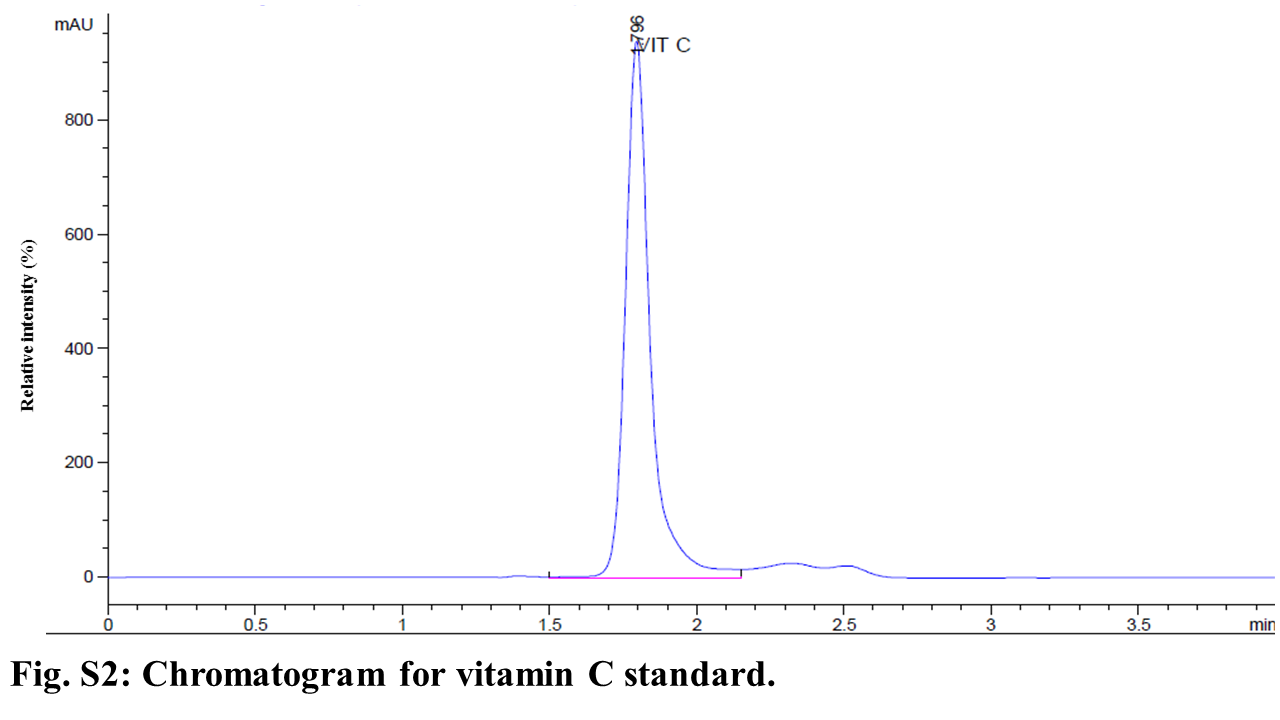

Supplement: Supplementary file 2 [file Image_2.tif]

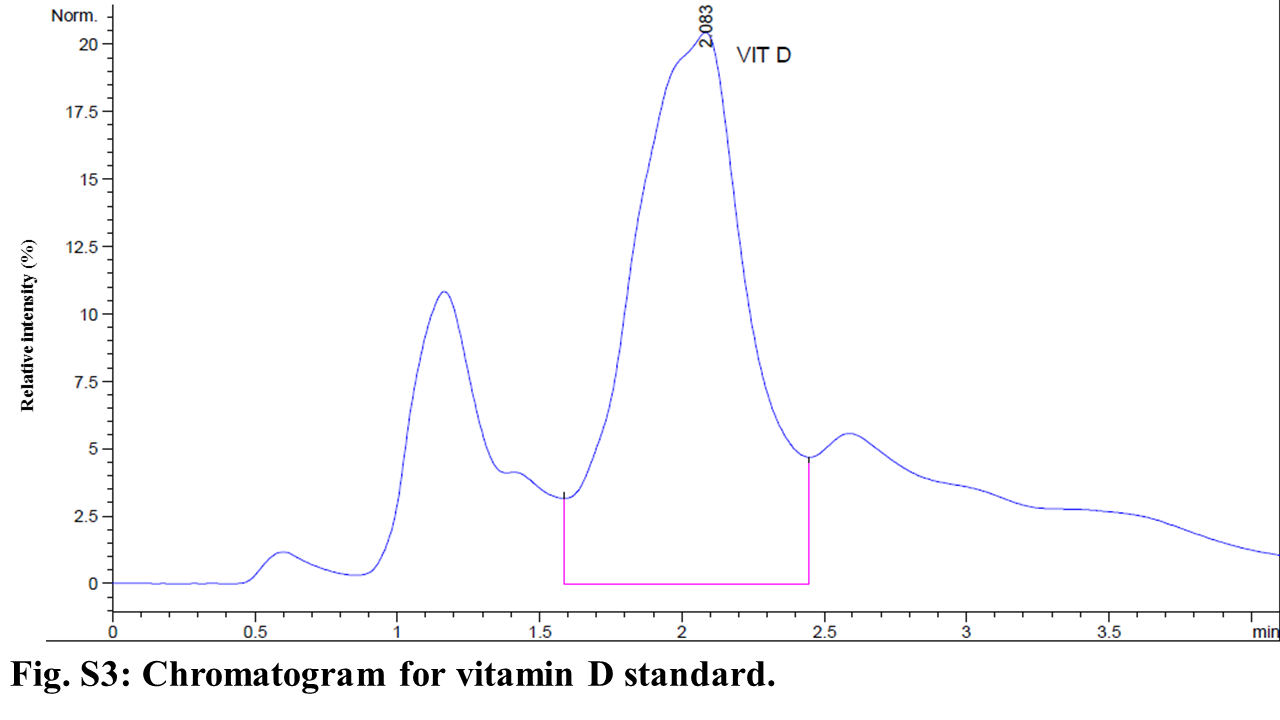

Supplement: Supplementary file 3 [file Image_3.tif]

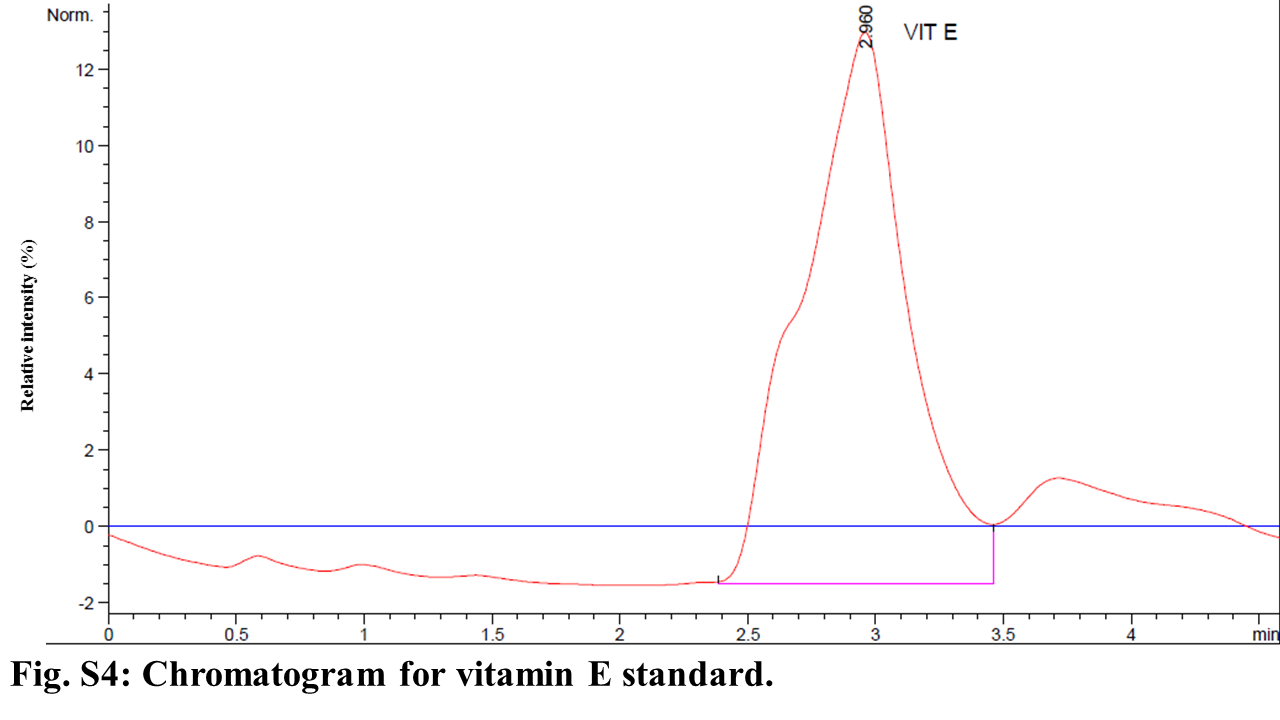

Supplement: Supplementary file 4 [file Image_4.tif]
